# Excessive daytime sleepiness and antipathogen drug consumption in the elderly: a test of the immune theory of sleep

**DOI:** 10.1038/srep23574

**Published:** 2016-03-21

**Authors:** Claire Berticat, Frédéric Thomas, Yves Dauvilliers, Isabelle Jaussent, Karen Ritchie, Catherine Helmer, Christophe Tzourio, Michel Raymond, Sylvaine Artero

**Affiliations:** 1 Institute of Evolutionary Sciences, University of Montpellier, Montpellier, France. (CNRS UMR 5554, IRD, EPHE); 2 IRD, MIVEGEC, (UMR CNRS/IRD/), Montpellier Cedex 5, France; 3 Centre de Référence Maladies Rares Narcolepsie et Hypersomnie Idiopathique, Service de Neurologie, Unité des Troubles du Sommeil, Hôpital Gui-de-Chauliac, CHU Montpellier, France; 4 INSERM U1061, La Colombière Hospital, University of Montpellier, Montpellier, France; 5 Faculty of Medicine, Imperial College, St Mary’s Hospital, London, United Kingdom; 6 INSERM, ISPED, UMR897, University of Bordeaux, Bordeaux, France; 7 INSERM, Neuroepidemiology, UMR897,University of Bordeaux, Bordeaux, France

## Abstract

The evolutionary reasons for sleep remain controversial. The immune theory of sleep suggests that sleep is essential to the immune system, allowing organisms to allocate more energy to their immunity. This hypothesis was tested by exploring the links between excessive daytime sleepiness (EDS) and vulnerability to infectious diseases in a large (n = 9294) cohort of elderly individuals, with information on socio-demographics, daily habits, and medical characteristics. At the two-year and four-year follow-ups, we obtained individual data from the national healthcare insurance about all medications prescribed to the participants between 2001 and 2003 (n = 2865). We found an independent positive association between EDS and the consumption of some anti-pathogen drugs. This relationship was mostly explained by fungal and parasitic infections rather than by viral and bacterial ones. These results, although based on correlations, are consistent with the idea that EDS as a proxy of altered sleep quality/quantity may affect the efficiency of the immune system, and hence vulnerability to infections.

Sleep is widespread in the animal kingdom, being described in insects, fish, birds, and mammals^1^,^2^,^3^,^4^,^5^,^6^. From an evolutionary perspective, it is predicted that sleep must be associated to substantial benefits to outweigh the multiple costs that are associated with dormancy states. While it is relatively easy to identify several of these costs (e.g., no mating or foraging opportunities, vulnerability to insect vectors and/or predators, low ability to detect and respond to changing environmental conditions), the benefits are more difficult to determine. However, sleep deprivation is usually rapidly accompanied by highly detrimental health consequences, indicating that sleep is a strict necessity in a great number of species. Currently, the main theories regarding the function of sleep^7^ propose that it plays an important role in brain development, clearance, or repair^8^,^9^,^10^, and in consolidating memories and learning^11^. Sleep could also be beneficial because it maximizes energy saving when other activities bring no, or little, fitness benefits^12^,^13^. Alternatively, there is an increasing body of evidence that sleep is essential to the immune system, allowing organisms to allocate more energy to their immunity^14^,^15^. To function properly, the immune system indeed requires a significant proportion of our daily energy budget^16^. An efficient way of allocating more energy to the immune system is to place all the other functions in ‘rest mode’, in other words, put them to sleep. In accordance with this hypothesis, it is a common belief that sleep deprivation is linked to increased vulnerability to infections^17^,^18^,^19^ and that sleep duration usually increases in sick individuals^20^, which significantly accelerates their recovery^21^,^22^. A recent interspecific study on mammals also supports the immune theory of sleep: the more the organisms sleep, the better their immune system functions and the fewer parasites they have^23^. This immunity hypothesis for the role of sleep still needs to be explored, particularly at the intraspecific level.

Here, we tested the immune theory of sleep in humans by exploring the links between excessive daytime sleepiness (EDS) and vulnerability to infectious diseases. We focused our study on elderly individuals because the effects of sleep changes are expected to be especially elevated in populations at particular risk of infections, like older adults^14^. In addition, aging is often associated with a decrease in both the quality and quantity of sleep, with large variations among individuals^24^,^25^,^26^,^27^, and EDS is frequently reported in the elderly^28^,^29^. To estimate changes in sleep quality/quantity, we focused on the complaint of EDS because although subjective it represents a unique relative individual estimate of sleep alteration whatever the total daily amount of time spent sleeping. Thus we use EDS in this analysis as a proxy of altered sleep quality/quantity. Using a four-year longitudinal study based on an elderly population, we examined whether EDS was associated with the consumption of anti-pathogen drugs.

## Results

### Baseline characteristics

The present analysis was carried out on 2,865 subjects 1,108 males and 1,757 females from three centres (Bordeaux, Toulouse, Montpellier; 31% of the subjects initially recruited at baseline) for whom all data were available, including follow-up data for medications and who did not have dementia or narcolepsy. The ages of the subjects in 2000 ranged from 65 to 93 years, with an average of 73.2 ± 4.9 years (Table 1). Overall 54% (1,538/2,865) reported having EDS (58% of men [647/1,108] and 51% of women [891/1,757]; Table 1). The mean consumption of the different types of drugs from CNAM-TS is detailed in Table 1.

### Relationship between EDS and anti-infectious medicine consumption

The relationship between excessive daytime sleepiness and drug consumption for the different models fitted is provided in Table 2. The quantity of antibiotics taken was not significantly linked to EDS (χ^2^ = 0.92, *df* = 1, *P* = 0.34; 15% of the variance was explained by the model). Similarly, no significant association between EDS and the frequency of antiviral consumption was detected (χ^2^ = 1.86, *df* = 1, *P* = 0.17; 5% of the variance was explained by the model). However, EDS was significantly associated with an increased frequency of antifungals consumption (χ^2^ = 5.33, *df* = 1, *P* = 0.02; 5% the variance was explained by the model) and the frequency of antiparasitics consumption (χ^2^ = 4.43, *df* = 1, *P* = 0.03; 9% of the variance was explained by the model). The sum of these four categories of anti-infectious drugs was not significantly influenced by EDS (χ^2^ = 0.77, *df* = 1, *P* = 0.38; 16.5% of the variance was explained by the model). This was probably largely caused by the antibiotics category, which represents 79% of all anti-infectious drugs consumed. Interestingly, the quantity of all non-infectious and non-psychotropic drugs was not significantly influenced by EDS (χ^2^ = 0.86, *df* = 1, *P* = 0.35; 16% of the variance was explained by the model). See the Supplementary Information for details on all regression models (Tables S2–S7).

## Discussion

We detected a positive relationship between the consumption of some anti-pathogen drugs and excessive daytime somnolence, which is consistent with the idea that sleep deprivation may affect immune system efficiency and hence vulnerability to infections^15^. The correlations were significant for parasitic and fungal infections but not for bacterial and viral infections.

Several hypotheses could be invoked to explain these findings. First, it is possible that sleep deprivation increases vulnerability to all infectious agents, but that depending on the pathogens considered, drug consumption may not be an entirely reliable proxy of the real infection frequency. For instance, while people are frequently infected by viruses, anti-viral drug consumption is low because it is rarely effective, e.g., the common cold and influenza are usually not treated with antiviral drugs. The correlation is presumably also weak with bacterial infections because antibiotics are often overprescribed^30^, thereby weakening the correlation between real bacterial infection rates and antibacterial drug consumption. However, pathologies due to fungal or parasitic infections are more specific, leading infected people to more systematically consult physicians and obtain targeted drugs^31^. Interestingly, prescribed antifungals were mainly (70%) dermatological, suggesting that there was no relationship here between consumption of antibiotics and antifungals^32^. The lack of a significant correlation between EDS and viral/bacterial infections could also suggest that the immune state is a rather poor predictor of the infection outcome when in contact with those pathogenic agents, that is, people with a poor immunity are not the only ones to get sick when in contact with a virus.

This study is strengthened by the large sample size, the population-based design, the four-year follow-up, and the adjustment with a wide range of covariates. However, we cannot exclude several alternative explanations, including a reverse causality, where somnolence would result from the detrimental effects of frequent infections on sleep quality/quantity. For instance, while influenza viruses increase sleep duration during the symptomatic period, they may conversely reduce it during the incubation period^33^. Several infections are also known to directly induce daytime somnolence without necessarily altering sleep quality itself^14^. We do not currently favour hypotheses suggesting that infections (indirectly or directly) cause somnolence, because daytime sleepiness most likely reflects a chronic rather than acute problem. Consequently, as suggested by the absence of correlation between consumption of anti-infectious drugs and EDS at baseline, even frequent infection episodes may remain too infrequent over the survey period to influence the self-rated level of somnolence of participants.

Certain illnesses, like depression, diabetes and chronic organic diseases, can also affect both sleep and immunity, and could then potentially lead to a spurious correlation between these two variables. Nevertheless, our results do not change even after adjusting for these potential confounding elements. As people get older, they have comorbid conditions more frequently and particularly high rates of depressive symptoms leading to immunosenescence^34^. In our study, the effects of these comorbidities were controlled. We cannot exclude that there is no infectious specificity in the response detected since daytime sleepiness is significantly associated with a wide range of lethal disorders in the elderly^35^,^36^,^37^.

Finally there are several limitations in this study. First all the results presented here are only correlations. Second, the infection was qualitatively detected by the presence of treatments, although its severity could not be quantitatively estimated. Thus, low grade infections, generally without specific treatments, could not be considered here. Despite extensive adjustments for socio-demographic and lifestyle factors, chronic diseases, and sleep medication, we cannot exclude the possibility that unmeasured confounding factors may explain part of any association detected in observed data. The assessment of sleep complaints was self-reported, and that remains the most common method for initial diagnosis and management in the primary healthcare setting. The measurement of EDS in this study was based on only one question, but its severity was examined using a four-point scale. However, an assessment of sleep apnea was not available, and the possibility remains that our results on EDS may be driven by these variables as we also hypothesized for sleep restriction per se.

Finally, sleep disturbance was assessed only once (at the baseline examination). We therefore could not assess the evolution of sleep disturbances in relation to antipathogen medication intake and thus determine whether sleep problems were stable, decreased, or increased in parallel with the drug intake.

Further long-term studies are needed to extend these findings and explore the role of EDS and sleep alteration in immunity and its consequences on the probabilities of specific infections. This is especially important in humans who suffer from sleep restriction and non-restorative sleep; over recent decades, these have been increasingly recognized as a public-health preoccupation for both individuals and the population as a whole^14^. A possible explanation for the link between EDS, sleep quality/quantity and the immune state is that change in the sleep profile may alter molecular processes that drive cellular immune activation and induce inflammatory cytokines^15^,^38^. Unraveling the molecular and cellular pathways by which sleep and the immune system are interrelated also appears to be a promising direction for understanding how sleep alterations could be more beneficial to some pathogens than others.

This question is of considerable public health interest given the high prevalence and secondary complication of infection in the elderly.

## Materials and Methods

### Participants

Between 1999 and 2001, non-institutionalized subjects were recruited as part of a multisite cohort study (Three City study, or 3C) of subjects aged at least 65 years old who were randomly selected from the electoral roll of three French cities (Bordeaux, Dijon, and Montpellier). Health-related data were collected during face-to-face interviews using standardized questionnaires. For the details of the study protocol, see^39^. A total of 9,294 subjects were included in the study (4,931 from Dijon, 2,104 from Bordeaux, and 2,259 from Montpellier). The study protocol was approved by the ethical committee of the university hospital of Kremlin-Bicêtre. The methods were carried out in accordance with the approved guidelines. Each participant signed legal consent forms.

The present study uses data collected at baseline (1999–2000) and at the two-year (2001–2002) and four-year (2003–2004) follow-up examinations for medication.

### Sleep complaint

At baseline, we defined EDS as the self-report of having a feeling of being excessively sleepy during the day. Participants were invited to answer ‘never, rarely, frequently, or often’ to the question, ‘Do you feel very sleepy during the day?’. Other information related to sleep were also recorded, including the prescription of hypnotic, antidepressant, and anxiolytic medications due to their action on sleep per se and snoring. Insomnia was also recorded; it was defined as the number of insomnia complaints self-reported when participants answered the following questions: ‘Do you have any difficulty in falling asleep?’ (difficulty in initiating sleep), ‘Do you wake up during the night?’(difficulty in maintaining sleep), ‘Do you often wake up early in the morning without being able to go back to sleep?’(early morning awakening)^28^,^40^.

### Medication

About half of the 3C cohort participants were affiliated with the French national health-care insurance for active or retired salaried workers (Caisse Nationale d’Assurance Maladie des Travailleurs Salariés; CNAM-TS). For these persons, we obtained individual data from the CNAM-TS about all drugs prescribed between 2001 and 2003. Drug names were coded according to the Anatomical Therapeutic Chemical classification of the World Health Organization and classified into one of five categories: antibiotics, antivirals, antifungals, antiparasitics, or non-infectious and non-psychotropic drugs (anxiolytics, hypnotics, and antidepressants) (see Supplementary Table S1). In this study, antipathogen drug consumption was considered as a proxy of infection rate and hence of immune system efficacy.

### Baseline data collection and examination

Data were collected by training nurses and psychologists during face-to-face interviews using standardized questionnaires at baseline (1999–2000). For the purpose of our study, baseline control variables were selected that included socio-demographic characteristics (age, sex, educational level, income, and if they were living alone), weight and height, and daily habits such as smoking and alcohol, coffee, and tea consumption. Depressive symptoms were defined by a score ≥16 on the Center for Epidemiological Studies Depression scale questionnaire^41^. Past history of cardiovascular heart disease included a history of angina pectoris, myocardial infarction, or revascularization procedures. Diabetes was defined as a fasting blood glucose level ≥7 mmol/l and/or drug treatment for diabetes. Blood pressure was measured twice with a digital electronic tensiometer. Information about asthma was also recorded.

A diagnosis of dementia was made by a neurologist according to DSM-IV^42^ criteria and validated by an independent national panel of neurologists. Mild Cognitive Impairment (MCI) (which is considered a prodrome to dementia) was diagnosed according to the currently used revised criteria (MCI-R) proposed by an international consensus group^43^. The detailed MCI definition has been reported elsewhere^44^. Verbal neurocognitive functioning and premorbid IQ estimate were assessed through the National Adult Reading Test (NART)^45^. All medication used during the previous month were recorded by interviewers. Participants were asked to show medical prescriptions and drug packages. Drug names were coded according to the Anatomical Therapeutic Chemical classification of the World Health Organization. There was no significant association between the consumption of anti-infectious drugs recorded at baseline and EDS, suggesting that these two variables were not directly influencing each other at baseline (Fisher exact tests; antibiotics: *P* = 0.32, antivirals: *P* = 0.33, antifungals: *P* = 0.23, antiparasitics: *P* = 0.44).

### Statistical analyses

The quantity of each anti-infectious drug category (antibiotics, antivirals, antifungals, and antiparasitics) was considered as a dependent variable in a regression, with EDS (two levels: never, at least one) as the explanatory variable. The quantity of antibiotics followed a left-bounded error distribution (antibiotics consumption cannot be negative), thus a censored regression models (Tobit) was used. For the other drug categories, consumption was pooled into two categories (two levels: never, at least one), and logistic regressions were used. The sum of all anti-infectious drugs was also considered as a dependent variable, and a Tobit regression model was used (anti-infectious drugs consumption cannot be negative). For all models, the control variable was the sum of all drugs consumed (CNAM-TS record), and the potential confounding variables were sampling location (three levels: Bordeaux, Dijon, Montpellier), sex (binary), age (quantitative), educational level (four levels), income (five levels), snoring level (four levels), smoking (binary), alcohol consumption (three levels: drinker, abstainer, ex-drinker), mg of caffeine per day (quantitative), asthma (binary), high blood pressure (binary), lifestyle (two levels: alone/not alone), past history of cardiovascular heart disease (binary), depressive symptoms (binary), diabetes (binary), MCI score (binary), NART score (six levels), BMI (or weight/height^2^, quantitative), consumption of anxiolytic, antidepressant, and hypnotic drugs from the interview (two levels: never, at least one), and insomnia (two levels: never/rarely; frequently/often). The interaction terms of sex and age with the dependent variables, educational level, income, MCI score, insomnia, consumption of anxiolytic, consumption of antidepressant, and consumption of hypnotic drugs were considered and removed when non-significant (see Supplementary Information for details). The quantity of all non-infectious and non-psychotropic drugs consumed was also considered as a dependent variable, with the same explanatory, control, and confounding variables as above. All statistical analyses were performed with R 2.11.1 software (www.r-project.org).

### Ethics Statement

The study protocol was approved by the ethical committee of the university hospital of Kremlin-Bicêtre. The methods were carried out in accordance with the approved guidelines. Each participant signed legal consent forms. Informed consent was obtained from all subjects.

## Additional Information

**How to cite this article**: Berticat, C. *et al.* Excessive daytime sleepiness and antipathogen drug consumption in the elderly: a test of the immune theory of sleep. *Sci. Rep.* **6**, 23574; doi: 10.1038/srep23574 (2016).

## Supplementary Material

Supplementary Information

## Figures and Tables

**Table 1:** Baseline characteristics of the study sample.

Sex	Age	Antivirals	Antiparasitics	Antifungals	Antibiotics	Non anti-infectious and psychotropic drugs	Complaint of EDS
	mean (SD)	n	n	n	mean (SD)	mean (SD)	n (%)
Men(N = 1108)	73.1(4.8)	42	58	382	3.7 (4.5)n = 916	120 (80)n = 1108	647 (58)
Women(N = 1757)	73.2(4.9)	112	95	668	4.1 (4.9)n = 1484	132 (79)n = 1757	891 (51)
All(N = 2865)	73.2(4.9)	154	153	1050	3.97 (4.7)n = 2400	127 (80)n = 2865	1,538 (54)

Sex, sample size (N), age, excessive daytime sleepiness (EDS), and consumption of the different types of medicines from 2001 to 2003 recorded from CNAM-TS. For the medicines, ‘n’ refers to the number of individuals taking at least one of that type of drug during the period considered. For the consumption of drugs described by a quantitative variable, the mean and standard deviation of the global distribution is given. For EDS, the number and the percentage of individuals reporting sleep deficit is indicated. See Methods for details.

**Table 2:** Influence of excessive daytime sleepiness (EDS) on consumption of drugs for the different models fitted, all things being equal (n = 2,865).

Explained variable of the model	Estimate of effect from EDS	SE	χ^2^	*df*	*P*(>χ^2^)
Anti-infectious (Tobit)	+0.20	0.27	0.55	1	0.46
Antibiotics (Tobit)	+0.19	0.20	0.92	1	0.34
Antivirals (Logistic)	+0.28	0.18	2.49	1	0.11
**Antifungals (Logistic)**	+**0.19**	**0.08**	**5.12**	**1**	**0.02**
**Antiparasitics (Logistic)**	+**0.39**	**0.19**	**4.40**	**1**	**0.03**
Non anti-infectious and psychotropics (Tobit)	−0.22	0.24	0.81	1	0.37

For each model, estimate of the effect of EDS relative to its absence, standard error (SE), χ^2^ statistic, degree of freedom (df), and *P*-value of the χ^2^ test are given. Items in bold showed significant effects.
