# Supplementary material for: Excessive daytime sleepiness and antipathogen drug consumption in the elderly: a test of the immune theory of sleep
Source: Sci Rep. 2016 Mar 21;6:23574. doi: 10.1038/srep23574 (PMC4800730; doi:10.1038/srep23574)
Supplement: Supplementary Information [file srep23574-s1.pdf]

Supplementary Information

Excessive daytime sleepiness and antipathogen drug consumption in the elderly: a test of the immune theory of sleep

Berticat Claire [1¶], Thomas Fréderic [2¶], Dauvilliers Yves[3, 4], Jaussent Isabelle[4], Ritchie Karen[4,5], Helmer Catherine[6], Tzourio Christophe[7], Raymond Michel[1], Artero Sylvaine [4*]

[¶] co-first authors

* Corresponding author

E-mail: sylvaine.artero@inserm.fr

Table S1. Classification of drugs. Classification of drugs according to the Anatomical Therapeutic Chemical classification of the World Health Organization into categories: antibiotics, antivirals, antifungals, antiparasitics, anxiolytics, hypnotics, antidepressants, and non-infectious and non-psychotropic drugs.

Drugs	ATC Classification
Antibiotics	A07AA-A07AB-A07-A07AX03-A07AX04-D06A-D06C-D07C-G01AA-G01AB-G01AC-G01AE-G01BA-G01BC-G01BD-G04AB-G04AC -G04AG-G04AH-J01-J04-S01AA-S01AB
Antivirals	J05-S01AD
Antifungals	A07AC-D01A-D01B-G01AF-G01AG-G01BE-G01BF-J02
Antiparasitics	A07AX01-A07AX02-P01-P02
Antidepressants	N06A
Anxiolytics	N05B
Antihypnotics	N05C
Non-infectious and non-psychotropic drugs	A01-A02-A03-A04-A05-A06 -A09-A10-A11-A12-A13-A16-B-C-D02-D03-D04-D05-D06B-D07A-D07B-D11A-G01AD-G01AX-G02-G03-G04AA-G04AD-G04BD-G04C-H-J06-J07-L-M-N01-N02-N03-N04-N05A-N06B-N06C-N06D-N07-P03-R-S01AX-S01B-S01C-S01E-S01F-S01G-S01H-S01J-S01K-S01X-S02-S03-V

Table S2. Tobit regression model of the quantity of antibiotics consumed as a function of excessive daytime sleepiness (EDS), totality of medicines taken, and potential confounding variables (n=2,865). For each variable, the estimate, standard error of the mean (SE), *P*-value of the Student's test of the mean, χ^2 statistic (Likelihood Ratio Test), and *P*-value of the χ^2 test are given. For categorical variables, the estimates are for one category compared to the reference category (underlined term). Items in bold showed significant effects.

	Estimate	SE	*P*(>|t|)	χ^2	*P*(>χ^2)
Intercept	7.28	6.33	0.25		
EDS	0.19	0.20	0.38	0.92	0.34
Totality of drugs	0.02	0.0015	$<10^{-5}$	305	**$<10^{-5}$**
Insomnia (frequently/<u>rarely</u>)	0.18	0.2	0.38	0.77	0.38
Snoring				4.23	0.24
rarely/<u>never</u>	0.345	0.24	0.16		
regularly/<u>never</u>	-0.12	0.27	0.67		
frequently/<u>never</u>	0.35	0.33	0.29		
Age	0.07	0.08	0.41		
Sex	-0.24	0.42	0.57		
Body mass Index (BMI)	-0.05	0.024	0.04	4.38	**0.04**
Educational level (lower to higher)					
2/<u>1</u>	-1.07	0.42	0.01		
3/<u>1</u>	-0.61	0.49	0.21		
4/<u>1</u>	-0.35	0.50	0.48		
Income (1 to 4: lower to higher, 5: do not wish to reply)					
2/<u>1</u>	-2.66	6.64	0.69		
3/<u>1</u>	-7.58	6.77	0.26		
4/<u>1</u>	3.82	6.70	0.57		
5/<u>1</u>	-6.70	1.1	0.54		
National Adult Reading Test (NART) score (lower to higher)				14.64	**0.01**
2/<u>1</u>	-0.61	6.637	0.19		
3/<u>1</u>	-0.69	0.47	0.16		
4/<u>1</u>	-0.92	0.49	0.05		
5/<u>1</u>	-0.20	0.47	0.68		
6/<u>1</u>	-1.20	0.49	0.02		
Mild Cognitive Impairment :MCI (yes/<u>no</u>)	0.04	0.20	0.85	0.04	0.85
Lifestyle (not alone/<u>alone)</u>	0.33	0.25	0.19	1.74	0.19
Alcohol consumption				0.54	0.76

drinker/abstainer	0.006	0.26	0.98		
ex-drinker/abstainer	0.46	0.66	0.48		
History of cardiovascular disease (yes/no)	-0.57	0.34	0.10	2.73	0.10
Depressive symptoms (yes/no)	0.60	0.30	0.04	4.12	**0.04**
Smoker (yes/no)	0.24	0.23	0.30	1.05	0.30
mg of caffeine per day	0.0009	0.0006	0.16	1.97	0.16
Asthma (yes/no)	1.59	0.37	$<10^{-5}$	18.5	**$<10^{-5}$**
High blood pressure (yes/no)	-0.85	0.21	$<10^{-5}$	15.9	**$<10^{-5}$**
Diabetes (yes/no)	0.38	0.35	0.28	1.16	0.28
hypnotic drugs (yes/no)	0.05	0.33	0.88	0.02	0.88
Anxiolytic drugs (yes/no)	0.12	0.28	0.65	0.20	0.65
Antidepressant drugs (yes/no)	-12.3	5.53	0.03		
Centre				0.30	0.86
Dijon/Bordeaux	-0.09	0.28	0.72		
Montpellier/Bordeaux	0.03	0.33	0.93		
Sex*Educational level				8.32	**0.04**
female/male*2/1	0.95	0.5	0.06		
female/male*3/1	0.91	0.59	0.12		
female/male*4/1	-0.07	0.64	0.91		
Age * Income				10.15	**0.04**
Age*2/1	0.03	0.09	0.74		
Age*3/1	0.10	0.09	0.25		
Age*4/1	-0.05	0.09	0.25		
Age*51	0.09	0.15	0.53		
Age*Antidepressant drugs (yes/no)	-0.16	0.07	0.03	4.80	**0.03**

Table S3. GLM with error binomial predicting the quantity of antiviral drugs consumed as a function of excessive daytime sleepiness (EDS) and potential confounding variables (n=2,865). For each variable, the estimate, standard error of the mean (SE), *P*-value of the Student's test of the mean, χ^2 statistic, and *P*-value of the χ^2 test are given. For categorical variables, the estimates are for one category compared to the reference category (underlined term).

	Estimate	SE	$P(>\lvert t\rvert)$	χ^2	$P(>\chi^2)$
Intercept	1.09	1.78	0.54		
EDS	0.28	0.18	0.12	2.49	0.11
Totality of drugs	0.004	0.0011	$<10^{-3}$	0.02	0.88
Insomnia (frequently/rarely)	0.02	0.18	0.88	0.02	0.87
Snoring				0.008	0.93
rarely/never	-0.18	0.21	0.40		
regularly/never	-0.08	0.24	0.73		
frequently/never	0.07	0.28	0.81		
Age	-0.05	0.02	0.01		
Sex	0.43	0.24	0.07	3.55	0.06
Body Mass Index (BMI)	-0.06	0.02	0.007	7.38	0.006
Educational level (lower to higher)				4.44	0.22
2/1	0.06	0.22	0.78		
3/1	-0.33	0.32	0.30		
4/1	0.33	0.32	0.30		
Income (1 to 4: lower to higher, 5: do not wish to reply)				3.9	0.42
2/1	0.36	0.43	0.39		
3/1	0.17	0.46	0.71		
4/1	-0.08	0.48	0.86		
5/1	0.51	0.63	0.42		
National Adult Reading test (NART) score (lower to higher)				1.13	0.95
2/1	-0.14	0.39	0.72		
3/1	-0.39	0.43	0.37		
4/1	-0.19	0.40	0.63		
5/1	-0.15	0.42	0.71		
6/1	-0.11	0.43	0.80		
Mild Cognitive Impairment (MCI) (yes/no)	-0.11	0.18	0.54	0.32	0.57
Lifestyle (not alone/alone)	-0.05	0.22	0.81	0.04	0.84
Alcohol consumption				8.94	0.01
drinker/abstainer	0.52	0.25	0.04		
ex-drinker/abstainer	-1.15	1.04	0.27		
History of cardiovascular disease (yes/no)	-0.13	0.32	0.69	0.16	0.68
Depressive symptoms (yes/no)	-0.16	0.26	0.54	0.32	0.57
smoker (yes/no)	-0.10	0.21	0.61	0.25	0.62
mg of caffeine per day	-0.0001	0.0006	0.82	0.04	0.84

Asthma (yes/no)	0.04	0.31	0.89	0.02	0.87
High blood pressure (yes/no)	0.0001	0.19	0.99	$<10^{-5}$	0.99
Diabetes (yes/no)	-0.22	0.35	0.53	0.48	0.49
Antihypnotic drugs (yes/no)	0.29	0.26	0.26	1.22	0.27
Anxiolytic drugs (yes/no)	-7.8	3.23	0.01		
Antidepressant drugs (yes/no)	0.22	0.33	0.50	0.45	0.50
Centre				0.60	0.74
Dijon/Bordeaux	0.15	0.26	0.55		
Montpellier/Bordeaux	0.23	0.30	0.45		
Age*Anxiolytic drugs (yes/no)	0.1060	0.04686	0.023718		

Table S4. GLM with error binomial predicting the quantity of antifungal drugs consumed as a function of excessive daytime sleepiness (EDS) and potential confounding variables (n=2,865). For each variable, the estimate, standard error of the mean (SE), P-value of the Student's test of the mean, χ^2 statistic, and P-value of the χ^2 test are given. For categorical variables, the estimates are for one category compared to the reference category (underlined term). Items in bold showed significant effects.

	Estimate	SE	*P*(>|t|)	χ^2	*P*(>χ^2)
Intercept	-0.38	0.82	0.64		
**EDS**	**0.19**	**0.08**	**0.02**	**5.12**	**0.02**
Totality of drugs	0.006	0.00067	$<10^{-5}$	103.6	$<10^{-5}$
Insomnia (frequently/rarely)	0.008	0.09	0.92	0.006	0.94
Snoring				0.26	0.61
rarely/never	0.06	0.10	0.56		
regularly/never	-0.10	0.12	0.39		
frequently/never	-0.006	0.14	0.96		
Age	-0.02	0.008	0.03	4.98	0.02
Sex	-0.52	0.38	0.18		
Body Mass Index (BMI)	0.02	0.01	0.02	5.21	0.02
Educational level (lower to higher)				5.97	0.11
2/1	-0.09	0.11	0.37		
3/1	0.07	0.14	0.60		
4/1	0.25	0.16	0.12		
Income (1 to 4: lower to higher, 5: do not wish to reply)				5.50	0.24
2/1	-0.31	0.20	0.12		
3/1	-0.23	0.21	0.28		
4/1	-0.09	0.22	0.68		
5/1	-0.06	0.31	0.84		
National Adult Reading (NART) score (lower to higher)					
2/1	-0.27	0.32	0.39		
3/1	-0.37	0.33	0.27		
4/1	-0.12	0.31	0.69		
5/1	-0.50	0.33	0.13		
6/1	-0.36	0.33	0.27		
Mild Cognitive Impairment (MCI) (yes/no)	0.32	0.14	0.02		
Lifestyle (not alone/alone)	-0.13	0.10	0.22	1.51	0.22
Alcohol consumption				2.07	0.35
drinker/abstainer	0.16	0.11	0.16		
ex-drinker/abstainer	0.22	0.27	0.42		
History of cardiovascular disease (yes/no)	-0.51	0.15	$<10^{-3}$	11.64	$<10^{-3}$

Depressive symptoms (yes/no)	0.04	0.12	0.74	0.10	0.75
Smoker (yes/no)	-0.09	0.09	0.37	0.79	0.37
mg of caffeine per day	0.0002	0.0003	0.53	0.35	0.56
Asthma (yes/no)	0.13	0.15	0.41	0.64	0.42
High blood pressure (yes/no)	-0.23	0.09	0.01	6.20	0.01
Diabetes (yes/no)	0.0007	0.15	0.99	8.10^{-5}	0.99
Antihypnotic drugs (yes/no)	0.05	0.14	0.70	0.15	0.70
Anxiolytic drugs (yes/no)	0.06	0.12	0.62	0.23	0.63
Antidepressant drugs (yes/no)	-0.08	0.18	0.65	0.19	0.66
Centre				0.48	0.79
Dijon/Bordeaux	0.06	0.12	0.64		
Montpellier/Bordeaux	0.02	0.14	0.89		
Sex (female/male)*NART score				11.85	0.04
female/male*2/1	1.00	0.41	0.01		
female/male*3/1	0.91	0.43	0.03		
female/male*4/1	0.54	0.40	0.17		
female/male*5/1	1.13	0.41	0.006		
female/male*6/1	0.71	0.41	0.08		
Sex (female/male)*MCI	-0.36	0.17	0.03340	4.55	0.03

Table S5. GLM with error binomial predicting the quantity of antiparasitic drugs consumed as a function of excessive daytime sleepiness (EDS) and potential confounding variables (n=2,865). For each variable, the estimate, standard error of the mean (SE), P-value of the Student's test of the mean, χ^2statistic, and P-value of the χ^2 test are given. For categorical variables, the estimates are for one category compared to the reference category (underlined term). Items in bold showed significant effects.

	Estimate	SE	*P*(>|t|)	χ^2	*P*(>χ^2)
Intercept	0.55	1.82	0.76		
EDS	**0.39**	**0.19**	**0.040**	**4.40**	**0.03**
Totality of drugs	0.006	0.0012	$<10^{-5}$	27.8	$<10^{-5}$
Insomnia (frequently/rarely)	0.17	0.19	0.36	0.83	0.36
Snoring				5.12	0.16
Rarely/never	0.007	0.23	0.978		
Regularly/never	0.46	0.24	0.06		
Frequently/never	0.016	0.30	0.96		
Age	-0.05	0.02	0.01		
Sex	-2.15	0.90	0.02		
Body Mass Index (BMI)	0.002	0.02	0.92	0.008	0.92
Educational level (lower to higher)				5.52	0.14
2/1	0.30	0.25	0.22		
3/1	0.61	0.30	0.04		
4/1	0.69	0.34	0.04		
Income (1 to 4: lower to higher, 5: do not wish to reply)				1.05	0.90
2/1	-0.03	0.44	0.94		
3/1	0.04	0.46	0.93		
4/1	0.22	0.48	0.65		
5/1	0.03	0.75	0.96		
National Adult Reading test (NART) score (lower to higher)					
2/1	-0.49	0.58	0.40		
3/1	-0.79	0.63	0.21		
4/1	-0.79	0.57	0.17		
5/1	-0.68	-0.60	0.26		
6/1	-1.78	0.71	0.01		
Mild Cognitive Imparment (MCI) (yes/no)	-0.04	0.18	0.81	0.06	0.81
Lifestyle (couple/alone)	0.23	0.22	0.29	1.10	0.29
Alcohol consumption				4.88	0.09
drinker/abstainer	-0.50	0.22	0.03		
ex-drinker/abstainer	-0.16	0.53	0.75		
History of cardiovascular disease (yes/no)	-0.06	0.29	0.83	0.05	0.83
Depressive symptoms (yes/no)	-0.09	0.36	0.72	0.13	0.72

Smoker (yes/no)	-0.35	0.21	0.10	2.73	0.10
mg of caffeine per day	$9.6\ 10^{-5}$	$6.1\ 10^{-4}$	0.87	0.02	0.88
Asthma (yes/no)	-0.34	0.35	0.33	1.01	0.31
High blood pressure (yes/no)	-0.45	0.19	0.02	5.33	0.02
Diabetes (yes/no)	-0.20	0.32	0.54	0.39	0.53
Antihypnotic drugs (yes/no)	0.54	0.24	0.03		
Anxiolytic drugs (yes/no)	0.70	0.38	0.07		
Antidepressant drugs (yes/no)	-10.2	4.30	0.02		
Centre				17.74	$<10^{-3}$
Dijon/Bordeaux	0.02	0.26	0.93		
Montpellier/Bordeaux	0.90	0.28	0.001		
Sex (female/male)*NART score				17.62	0.003
female/male*2/1	1.16	0.10	0.24		
female/male*3/1	1.98	0.99	0.05		
female/male*4/1	2.03	0.94	0.03		
female/male*5/1	2.05	0.95	0.03		
female/male*6/1	2.91	1.02	0.004		
Sex (female/male)*Anxiolytic drugs (yes/no)	-0.96	0.48	0.04	4.32	0.04
Sex (female/male)*Antidepressant drugs (yes/no)	2.21	1.18	0.06	5.47	0.02
Age*Antidepressant drugs (yes/no)	0.11	0.05	0.04	5.0	0.02

Table S6. Tobit regression model of the quantity of anti-infectious drugs consumed as a function of excessive daytime sleepiness (EDS), totality of medicines taken, and potential confounding variables (N=2.865). For each variable, the estimate, standard error of the mean (SE), p-value of the Student's test of the mean, χ^2 statistic (Likelihood Ratio Test), and P-value of the χ^2 test are given. For categorical variables, the estimates are for one category compared to the reference category (underlined term).

	Estimate	SE	*P*(>|t|)	χ^2	*P*($>\chi^2$)
Intercept	3.95	8.24	0.63		
EDS	0.20	0.27	0.46	0.55	0.46
Totality of drugs	0.04	0.002	$<10^{-5}$	369	$<10^{-5}$
Insomnia (frequently/rarely)	0.09	0.27	0.74	0.09	0.75
Snoring				2.76	0.10
Rarely/never	0.49	0.32	0.12		
Regularly/never	0.17	0.36	0.64		
Frequently/never	0.91	0.43	0.03		
Age	-0.04	0.11	0.68		
Sex	0.51	0.34	0.13	2.23	0.13
Body Mass Index (BMI)	-0.04	0.03	0.21	1.71	0.19
Educational level (lower to higher)				5.51	0.14
2/1	-0.74	0.33	0.03		
3/1	-0.19	0.43	0.66		
4/1	-0.41	0.49	0.40		
Income (1 to 4: lower to higher, 5: do not wish to reply)					
2/1	2.23	8.72	0.80		
3/1	-5.27	8.88	0.55		
4/1	13.42	8.81	0.13		
5/1	-1.12	14.45	0.94		
National Adult reading test (NART) score (lower to higher)				13.12	0.02
2/1	0.10	0.62	0.87		
3/1	-0.04	0.66	0.94		
4/1	-0.33	0.62	0.59		
5/1	0.73	0.65	0.26		
6/1	-0.61	0.68	0.37		
Mild Cognitive Impairment (MCI) (yes/no)	0.16	0.27	0.55	0.33	0.56
Lifestyle (couple/alone)	0.29	0.33	0.38	0.78	0.37
Alcohol consumption				0.90	0.64
drinker/abstainer	0.34	0.35	0.33		
ex-drinker/abstainer	0.29	0.87	0.74		
History of cardiovascular disease (yes/no)	-1.41	0.46	0.002	9.35	0.002

Depressive symptoms (yes/no)	0.84	0.39	0.03	4.46	0.03
Smoker (yes/no)	-0.06	0.31	0.85	0.03	0.86
mg of caffeine per day	6.10^{-4}	8.10^{-4}	0.48	0.43	0.51
Asthma (yes/no)	1.74	0.49	$< 10^{-3}$	12.6	$<10^{-3}$
High blood pressure (yes/no)	-1.17	0.28	$< 10^{-4}$	17.08	$<10^{-5}$
Diabetes (yes/no)	0.17	0.47	0.71	0.13	0.72
Antihypnotic drugs (yes/no)	0.26	0.43	0.55	0.34	0.56
Anxiolytic drugs (yes/no)	0.13	0.37	0.71	0.11	0.73
Antidepressant drugs (yes/no)	-0.35	0.56	0.53	0.38	0.54
Centre				0.82	0.66
Dijon/Bordeaux	0.03	0.37	0.94		
Montpellier/Bordeaux	0.30	0.43	0.49		
Age * Income				15.7	0.003
Age*2/1	-0.04	0.12	0.73		
Age*3/1	0.07	0.12	0.54		
Age*4/1	-0.17	0.12	0.13		
Age*51	-0.03	0.19	0.89		

Table S7. Tobit regression model of the quantity of all non-infectious drugs consumed as a function of excessive daytime sleepiness (EDS), totality of medicines taken, and potential confounding variables (n=2,865). For each variable, the estimate, standard error of the mean (SE), p-value of the Student's test of the mean, χ^2 statistic (Likelihood Ratio Test), and p-value of the χ^2 test are given. For categorical variables, the estimates are for one category compared to the reference category (underlined term).

	Estimate	SE	*P*(>|t|)	χ^2	*P*(>χ^2)
Intercept	-6.69	7.43	0.37		
EDS	-0.22	0.24	0.37	0.81	0.37
Totality of drugs	0.97	0.001	$<10^{-5}$	13000	$<10^{-5}$
Insomnia (frequently/rarely)	-0.09	0.24	0.72	0.11	0.73
Snoring				2.96	0.08
Rarely/never	-0.42	0.29	0.15		
Regularly/never	-0.17	0.33	0.59		
Frequently/never	-0.83	0.39	0.03		
Age	0.07	0.10	0.50		
Sex	-0.48	0.30	0.11	2.47	0.12
Body Mass Index (BMI)	0.04	0.03	0.17	2.06	0.15
Educational level (lower to higher)				4.76	0.19
2/1	0.63	0.30	0.04		
3/1	0.21	0.39	0.58		
4/1	0.23	0.44	0.60		
Income (1 to 4: lower to higher, 5: do not wish to reply)					
2/1	0.07	7.85	0.99		
3/1	6.65	8.01	0.41		
4/1	-9.12	7.94	0.25		
5/1	2.10	12.9	0.87		
National Adult Reading test NART score (lower to higher)				11.8	0.04
2/1	-0.12	0.56	0.83		
3/1	0.05	0.59	0.93		
4/1	0.34	0.56	0.55		
5/1	-0.60	0.59	0.30		
6/1	0.51	0.61	0.41		
Mild Cognitive Impairment (MCI) (yes/no)	-0.06	0.24	0.81	0.06	0.81
Lifestyle (couple/alone)	-0.26	0.29	0.38	0.79	0.37
Alcohol consumption				1.45	0.48
drinker/abstainer	-0.37	0.32	0.24		
ex-drinker/abstainer	-0.53	0.78	0.50		

History of cardiovascular disease (yes/no)	1.22	0.41	0.003	8.62	0.003
Depressive symptoms (yes/no)	-0.71	0.36	0.05	3.94	0.05
Smoker (yes/no)	0.08	0.28	0.76	0.08	0.77
mg of caffeine per day	-4.10^{-4}	8.10^{-4}	0.64	0.18	0.67
Asthma (yes/no)	-1.53	0.44	$<10^{-3}$	11.83	$<10^{-3}$
High blood pressure (yes/no)	0.93	0.25	$<10^{-3}$	13.58	$<10^{-3}$
Diabetes (yes/no)	-0.06	0.43	0.88	0.02	0.89
Antihypnotic drugs (yes/no)	-0.17	0.39	0.65	0.20	0.66
Anxiolytic drugs (yes/no)	-0.07	0.33	0.82	0.04	0.84
Antidepressant drugs (yes/no)	0.20	0.50	0.68	0.15	0.69
Centre				1.25	0.53
Dijon/Bordeaux	0.03	0.33	0.91		
Montpellier/Bordeaux	-0.30	0.39	0.44		
Age * Income				13.84	0.008
Age*2/1	0.006	0.10	0.95		
Age*3/1	-0.09	0.11	0.40		
Age*4/1	0.12	0.11	0.26		
Age*51	-0.04	0.17	0.81		
